# Observations on hearing preservation in patients with hybrid-L electrode implanted at Poznan University of Medical Sciences in Poland

**DOI:** 10.1007/s00405-012-2263-5

**Published:** 2012-12-07

**Authors:** Witold Szyfter, Maciej Wróbel, Michał Karlik, Łukasz Borucki, Maciej Stieler, Renata Gibasiewicz, Wojciech Gawęcki, Alicja Sekula

**Affiliations:** 1 Department of Otolaryngology and Oncological Laryngology, Poznan University of Medical Sciences, Ul. Przybyszewskiego 49, 60-355 Poznan, Poland; 2 Department of Phoniatrics and Audiology, Poznan University of Medical Science, Poznan, Poland

**Keywords:** Cochlear implant, Hybrid implant, Hybrid-L electrode, High frequency hearing loss, Hearing preservation

## Abstract

The objective of the paper is to evaluate the hearing preservation rate in patients with high frequency hearing loss, treated with Cochlear Nucleus Freedom Hybrid-L implant in the Otolaryngology Department, Poznan University of Medical Sciences in Poland. Study was designed as the retrospective analysis. Twenty-one patients were operated and implanted with Nucleus Freedom Hybrid-L implant. Pure tone thresholds were recorded prior to the surgery and at the time of speech processor switch-on. Patients were subdivided into two groups with respect to their PTA thresholds: group A—classic indications and group B—extended indications. Average PTA for three frequencies (250, 500, 1,000 Hz) were calculated for each patient pre- and postoperatively. In the group of 21 implanted patients in 17 cases we have observed preservation of hearing (12 patients from group A, 5 patients from group B) with a mean value of 13.1 dB. In 4 out of 21 patients deafness on the implanted ear was noted. Our results clearly indicate that with standard procedure hearing preservation can be obtained in majority of patients. Hearing preservation was not achieved in 19 %, but owing to design of the electrode of the Cochlear Nucleus Hybrid-L that enables to work as CI platform alone, in patients who lost their hearing after surgery re-implantations were not required. This proves that EAS is a safe and reliable method to help patients with specific type of hearing loss.

## Introduction

Selected group of patients with hearing loss at high frequency and still preserved hearing at low frequencies do not benefit from the classic hearing aid. Parallelly, due to residual hearing they do not meet the qualification criteria for classic cochlear implant. Nevertheless, ongoing development indicated that the hybrid system—concept of electro-acoustic stimulation [1] i.e., combination of hearing aid, that amplifies the residual hearing and cochlear implant, that stimulates cut off frequencies at the level of the basal turn of the cochlea will work best for that group of patients.

Preliminary observations that proceeded research and development on the hybrid system were combined with observations on conservation of residual hearing in cochlear implants recipients. Results showed that 50 % of operated patients retained sufficient hearing for effective use of ipsilateral ITE hearing aid [2]. Number of studies indicates that bimodal stimulation is not just a simple additive effect of both devices, but they show the synergistic action, that is especially appreciated by patients experiencing so called “cocktail party effect” in difficult listening environments [3–6].

Since then, a great progress has been made. New, less traumatic electrodes were introduced as well as the surgery improved from 1–1.2 mm cochleostomy to the round window approach. Atraumaticity i.e., preservation both hearing and vestibular function [7] become the key element in the hybrid surgery. Recent advances in that field brought the Hybrid-L electrode attached to Nucleus Freedom implant. Straight, thinner and shorter (16 mm) comparing with contour advanced electrode, but with 22 active contacts Hybrid-L electrode was developed to facilitate the less traumatic insertion through the round window to preserve residual hearing [8].

New Nucleus Freedom Hybrid-L system was introduced into the clinical practice in 2009. First experiences with new electrode were presented by Lenarz et al. [8] confirming the safety on both histological and clinical level [1, 9]. With that perspective authors would like to share their own observations on hearing preservation with hybrid-L system.

## Materials and methods

Study was designed as the retrospective analysis conducted at the Department of Otolaryngology and Oncological Laryngology, Poznan University of Medical Science. Twenty-one patients with high frequency hearing loss (*n* = 21) were included to the study. There were 12 females and 9 males, ranging in age from 16 to 77 years of age, with a mean of 49.5 years and median 52.5 years of age. Nucleus Freedom Hybrid-L device (Cochlear, Australia) was implanted in every patient by the same surgeon (WS) with the standardized surgical technique described previously [8, 10].

Patients were qualified for the surgery according to the audiometric indications (PTA), minimum 5 years of stable hearing loss, insufficient gain form hearing aid (30 % of speech understanding). Impressions for acoustic components were taken between 7th and 10th day after surgery. Speech processor switch-on was done in 5–8 weeks, depending on acoustic component delivery. Up to date, all the patients use electric as well as acoustic stimulation—none of the patient use only electric stimulation. Twelve patients use hearing aids contralaterally.

Studied cohort was divided into two subgroups with respect to preoperative PTA recordings. First one with classic indication for hybrid-L implant (group A, *n* = 13) and second with extended criteria according to Lenarz et al. (group B, *n* = 8) with residual hearing loss [1].

Audiometric results before and after surgery were analyzed. The mean value for three lower frequencies (250, 500 and 1,000 Hz) were calculated. Acoustic thresholds >110 dB or without recordings were considered as hearing loss and for statistic evaluation marked as 120 dB. The difference between mean values before and after surgery was studied and defined as follows: hearing preservation—mean value up to 10 dB; hearing impairment—mean value between 10 and 30 dB; hearing loss for patients with no thresholds recorded postoperatively.

## Results

In the group of 21 implanted patients in 17 cases we have observed preservation of hearing (12 patients from group A, 5 patients from group B). In 4 out of 21 patients deafness on the implanted ear was noted (group A—1 patient, group B—3 patients), although all these 4 patients reported significant difference in hearing after acoustic component activation.

In 17 patients with preservation of hearing analysis of audiometric results revealed that within the A group (13pts.) in 9 cases average hearing thresholds were calculated for 3 evaluated frequencies. In 3 cases hearing threshold for 1,000 Hz was not detectable, comparing to preoperative values: 80, 110 and 100 dB. The pre–post difference of mean hearing threshold was 0–10 dB in 6 cases, 11–20 dB in 3 cases, and 21–30 dB in 3 cases. Within the B group (8pts.) in 4 cases hearing threshold for three examined frequencies was established. The pre–post difference of mean hearing threshold was 0–10 dB in 3 cases and 21–30 dB in 2 cases. In one patient from the B group hearing threshold for 1,000 Hz was not recorded pre- and postoperatively (Table 1).

**Table 1:** Summary of the audiometric results in patients implanted with Nucleus Hybrid-L electrode recorded pre- and postoperatively

Patient number	Group	Implanted ear	Age	Gender	Pre-op PTA			Post-op PTA			Mean pre-op (dB)	Mean post-op (dB)	HL (dB)	Preservation of hearing
					250 Hz	500 Hz	1,000 Hz	250 Hz	500 Hz	1,000 Hz				
1	A	L	16.5	F	30	45	90	25	75	90	55.0	63.3	8.3	+
3	A	R	64	F	55	60	60	70	80	95	58.3	81.7	23.3	+
6	A	R	18.5	F	20	20	20	35	40	30	20.0	35.0	15.0	+
7	A	L	77	M	55	60	60	120	120	120	58.3	120.0	61.7	X
9	A	L	18	F	65	65	80	80	100	120	70.0	100.0	30.0	+
10	A	L	66	M	45	45	70	50	55	85	53.3	63.3	10.0	+
12	A	L	53.5	F	30	50	70	30	35	100	50.0	55.0	5.0	+
14	A	R	47	M	30	50	85	30	55	85	55.0	56.7	1.7	+
15	A	L	37	F	50	55	100	55	80	110	68.3	81.7	13.3	+
16	A	R	50	F	60	70	95	65	70	100	75.0	78.3	3.3	+
17	A	R	69	F	25	50	60	30	55	65	45.0	50.0	5.0	+
19	A	R	52.5	F	45	60	110	85	100	120	71.7	101.7	30.0	+
21	A	L	30.5	M	20	40	90	30	55	120	50.0	68.3	18.3	+
											Mean value for the group A^a^		13.6	
2	B	R	34.5	M	70	70	80	75	70	90	73.3	78.3	5.0	+
4	B	R	75.5	M	70	90	90	120	120	120	83.3	120.0	36.7	X
5	B	L	49.5	F	45	70	75	45	75	75	63.3	65.0	1.7	+
8	B	R	54.5	F	55	65	75	60	65	80	65.0	68.3	3.3	+
11	B	R	75	M	65	65	55	90	85	80	61.7	85.0	23.3	+
13	B	L	64	M	70	65	95	120	120	120	76.7	120.0	43.3	X
18	B	R	77.5	M	45	55	x	70	90	x	50.0	80.0	30.0	+
20	B	L	20.5	F	80	90	90	120	120	120	86.7	120.0	33.3	X
											Mean value for the group B^a^		12.7	
											Mean value for both groups (A + B) ^a^		13.1	
											Mean value for all patients in the study		19.1	

In all cases where threshold could not be recorded the value of 120 dB was used for statistic calculation

^a^Patient(s) with hearing loss excluded from the calculation

## Discussion

General overviews indicated that conservation of residual hearing in CI patient can be achieved in approximately 50 % [2, 11]. Years after first publication on the topic, Carlson et al. [12] interestingly pointed out that hearing preservation after standard cochlear electrode implantation remains unpredictable, but should be considered as the realistic goal. To improve the safety and increase the atraumaticity of the surgery various strategies were proposed, among which the technique called “soft surgery” proposed by Lehnhardt [13], become the gold standard in all CI surgeries. Other options, such as lubricants (i.e., hialuronic acid) added during the surgery are in the routine use [14], whereas, round window approach, as the alternative to the cochleostomy in classic CI remains under discussion [15].

Hybrid cochlear implants, unlikely the conventional ones, requires the residual hearing to give the recipient the best feedback. Therefore, hearing preservation is not an option, but the key point element to be achieved. First experiences with Hybrid S electrode (Cochlear, Australia) implanted in patients with residual hearing in low frequencies were very successful [16–19]. Although the design of the electrode (10 mm in length, six active contacts) was addressed to minimize trauma of the basilar turn of the cochlea, to reduce the risk of hearing loss, this could not be avoided. In patients with loss of residual hearing, the only electrical stimulation based on six contacts on the electrode was not sufficient, resulting in necessity of re-implantation with longer electrode.

Insertion of 16 mm Hybrid-L electrode has a standardized surgical procedure. This 16 mm with 22 contacts electrode covers approximately 270° of the basal turn of the cochlea, which is an equivalent of the former Nucleus 24 straight electrode. The stopper and the wing ascertains the comparable electrode position in the cochlea of the operated patients. Thus, variable such as insertion depth is not an issue in Cochlear Nucleus Freedom Hybrid-L implant. Data presented by Lenarz et al. [1] showed that all patients were successfully implanted through the round window. That is parallel with our experience, although, the position of the round window, caused minor technical problem during the insertion (the angle) and it was not the primary goal of investigation.

The overall hearing preservation rate in the current study was 80.95 % (17/21). Within the classic indication group (Group A) it was 92.3 % (12/13), but unfortunately, for patients with extended indications (group B) hearing preservation rate was lower—62.5 % (5/8). Nevertheless, it is still over 50 % above the level indicated in the literature. Looking at the potential cause of hearing loss in patients from our study, surgery variations was not an issue, because all cases were operated by the same surgeon. Thus, other reasons for the failure such as age of the patients (two were over 70 years), diabetes, vascular problems or inflammatory responses should be taken under consideration. These variables were not evaluated in the study.

## Conclusions

Our results clearly indicate that with standard procedure hearing preservation can be obtained in majority of patients. Implantations were successful in all cases with no major surgical problems. Hearing preservation was not achieved in 19.05 %, but owing to design of the electrode of the Cochlear Nucleus Hybrid-L that enables to work as CI platform alone, in patients who lost their hearing after surgery re-implantations were not required. This proves that EAS is a safe and reliable method to help patients with specific type of hearing loss.
